# Excess Weight in Relation to Lifestyle Habits in Spanish First-Year University Students: Differences between Pre- and Post-COVID-19—A Serial Cross-Sectional Study Based on uniHcos Project

**DOI:** 10.3390/healthcare11111547

**Published:** 2023-05-25

**Authors:** Natalia Hernández-Segura, Lorena Botella-Juan, Carmen Amezcua-Prieto, María Morales-Suárez-Varela, Ramona Mateos-Campos, Tania Fernández-Villa, Rocío Ortiz-Moncada, Ana Almaraz, Alicia Narciso-Rufo, Carlos Ayán-Pérez, Antonio José Molina, UniHcos Project Working Group

**Affiliations:** 1 Area of Preventive Medicine and Public Health, Department of Biomedical Sciences, Faculty of Health Sciences, Universidad de León, 24071 León, Spain; nhers@unileon.es (N.H.-S.); tferv@unileon.es (T.F.-V.); ajmolt@unileon.es (A.J.M.); 2 The Research Group in Gene-Environment and Health Interactions (GIIGAS), Institute of Biomedicine (IBIOMED), Universidad de León, 24071 León, Spain; 3 Consortium for Biomedical Research in Epidemiology & Public Health (CIBERESP), Carlos III Health Institute, Avenida Monforte de Lemos 3-5, Pabellón 11, Planta 0, 28029 Madrid, Spain; carmezcua@ugr.es (C.A.-P.); maria.m.morales@uv.es (M.M.-S.-V.); 4 Department of Preventive Medicine and Public Health, Universidad de Granada, 18016 Granada, Spain; 5 Instituto de Investigación Biosanitaria (ibs.Granada), 18014 Granada, Spain; 6 Research Group in Social and Nutritional Epidemiology, Pharmacoepidemiology and Public Health, Department of Preventive Medicine and Public Health, Food Sciences, Toxicology and Forensic Medicine, Faculty of Pharmacy, Universitat de València, Av. Vicent Andrés Estelles s/n, 46100 València, Spain; 7 Area of Preventive Medicine and Public Health, Department of Biomedical and Diagnostic Sciences, Universidad de Salamanca, 37007 Salamanca, Spain; 8 Area of Preventive Medicine and Public Health, Food and Nutrition Research Group (ALINUT), Universidad de Alicante, 03550 Alicante, Spain; rocio.ortiz@ua.es; 9 Department of Pathological Anatomy, Microbiology and Preventive Medicine and Public Health, School of Medicine, Universidad de Valladolid, 47005 Valladolid, Spain; aalmaraz@uva.es; 10 Centre for Research on Natural Resources, Health and Environment (RENSMA), Universidad de Huelva, 21071 Huelva, Spain; anarcisorufo@gmail.com; 11 Well-Move Research Group, Department of Special Didactics, University of Vigo, 36310 Vigo, Spain; cayan@uvigo.es

**Keywords:** overweight, exercise, students, university, COVID-19, public health, cross-sectional

## Abstract

The objective of this research was to study the relationship between the body weight and diet, physical activity, and other habits among freshmen students by sex, and to determine whether these habits have changed during the post-era of the COVID-19 pandemic. A serial cross-sectional study with data from 11 Spanish universities was carried out. In total, 10,096 first-year university students (73.2% female, mean age = 19.0 ± 1.5 years) completed an online self-administered questionnaire between 2012 and 2022. For some analyses, questionnaires were categorized by the year in which the survey was filled out as Before COVID-19, Lockdown, and New Normal. In total, 72.9% of participants were within the normal weight range, and 17.7% of men and 11.8% of women were overweight (*p* < 0.001). The students who did not meet the WHO criteria of physical activity, spent more than 7 h per day sitting, and skipped breakfast had a higher prevalence of obesity (*p* < 0.05). According to the period of study, the prevalence of overweight/obesity Before COVID-19 was 16.1% (95% CI: 15.4–16.9%), while in Lockdown the prevalence was significantly higher (20.2, 95% CI: 17.1–23.8) and in New Normal it was 18.9% (CI: 15.7–22.5). Moreover, the study suggests that during the Lockdown period, there was a reduction in the practice of physical activity and an increase in the prevalence of a healthy diet. For all these, it is necessary to propose public health interventions that improve the lifestyles of university students.

## 1. Introduction

Physical inactivity, a poor diet, and overweight/obesity are major risk factors for chronic conditions such as cardiovascular diseases, type 2 diabetes, cancer, and depression [1]. All these factors significantly impact the health status of university students who constitute a unique collective of particular relevance since they are the leaders, decision-makers, and parents of tomorrow [2]. Additionally, university students are in a critical period of their lives where they are transitioning into adulthood and forming lifelong habits. 

In this regard, systematic reviews have shown that the transition from high school to university is a critical period in which a decrease in physical activity levels is usually observed [3]. Similarly, during this transition, students eat less food, less healthy food, and less regularly [4]. These lifestyle changes can make university students susceptible to developing obesity [5]. Therefore, identifying and promoting healthy habits during this time can have a positive impact on their long-term health outcomes. Thus, a great deal of research on the prevalence of unhealthy habits has been carried out in this population with some of them showing a correlation between overweight/obesity and the diet, physical activity, or sedentary behaviour [6,7,8,9]. For instance, there is an increasing prevalence of obesity among college/university students in low-, middle-, and high-income countries [10]. This prevalence has been linked to health complications such as type 2 diabetes [11], increased serum uric acid concentration [12], or hypertension [10]. Thus, there is a need to encourage healthy habits that can help to avoid obesity-related complications among this population group, such as healthy food habits or physical activity. Indeed, university students show a positive attitude about preventing obesity through dieting and exercise [13]. However, studies have found low levels of physical activity among Spanish university students, with less than 30% of them considered to be sufficiently active [14].

Educational programs have been carried out to improve healthy habits among university students, with mixed results. For instance, Martínez-Rodríguez et al. [15] observed an improvement in body composition but not in the potential risk of developing an eating disorder after implementing a 4-month educational program. Similarly, interventions for promoting physical activity yielded inconsistent results, with limited evidence regarding both the immediate and the longer-term beneficial effects [16].

In general, university students show inadequate knowledge of healthy eating habits [17], which has also been linked to academic performance [18] or mental health (anxiety, stress, and depression) [19]. This also seems to be the case for physical inactivity, which is described as the inability to meet specific physical activity guidelines (e.g., 150–300 min of moderate intensity or 75–150 min of vigorous intensity physical activity per week, according to WHO (World Health Organization)) [20]. Physical inactivity is also prominent among university students, and it has been associated with quality of sleep [21], mental health [22], and academic performance [23]. 

This context worsened with the arrival of COVID-19 and the state of confinement it entailed. Several studies have showed that during the COVID-19 lockdown, rates of obesity, inadequate nutritional habits, and physical inactivity prevalence increased in this population [24,25,26]. As a result, a new scientific field of interest has emerged, specifically focused on the prevalence of these health markers in the post-COVID era [27].

There is another field of scientific interest within the university population that is represented by first-year university students. First-year university students (freshmen) constitute a population especially prone to developing poor lifestyle choices that lead to a significant increase in said risk factors [28]. During the transition from secondary school to university, freshmen students face a critical and vulnerable period for body weight changes, unhealthy eating, and physical inactivity, mainly due to being under high academic pressure, while having unprecedented freedom for selecting lifestyle choices living away from home [29]. Indeed, several studies have confirmed that freshmen tend to gain weight, practice unhealthy diet habits, and show a low motivation towards physical activity [30,31,32]. An under-investigated topic that requires further attention is to determine whether the prevalence of certain habits has changed among individuals who are starting their university studies as a result of COVID-19. More specifically, little is known about whether the lifestyle habits that prevailed among this group before the pandemic showed a similar trend again after the period of confinement.

An accurate approach to addressing this topic is needed for at least two reasons. Firstly, it would help to understand how university students have adapted to issues of health and well-being after experiencing a pandemic. The ability to adapt successfully to disturbances is often referred to as resilience [33]. Identifying factors of resilience among freshmen would help anticipate changes in healthy habits and educate them to maintain a healthy lifestyle. In addition, by identifying and promoting healthy habits in this population, health-related professionals and researchers can help prevent or reduce the incidence of negative health outcomes. This is a matter of concern, since university students are important agents of change, and promoting healthy habits among them can have a positive impact on future generations [34].

In light of all this, this research has a double-fold objective. In the first place, it aims to study the relationship between the body weight, diet, physical activity, and other habits among freshmen students. Second, it attempts to determine whether diet and physical activity habits have changed among freshmen students during the post-era of the COVID-19 pandemic.

## 2. Materials and Methods

### 2.1. Study Design and Sample

A serial cross-sectional study design was performed, based on the baseline survey of the uniHcos Project [35] collected between January 2012 and May 2022. In the interest of characterizing the young university population, obtaining a sample as homogeneous as possible, and reflecting the true lifestyle habits of freshmen students, participants older than 25 years were excluded (*n* = 1195, 10.4%). In addition, participants with missing or inconsistent data in any of the variables of interest analysed were eliminated (*n* = 194, 1.7%), so the final sample for the data analysis was N = 10,096 students.

Since the objective of the uniHcos Project is to create a dynamic university student cohort that collects data on different lifestyle habits of first-year university students and follow-up lifestyle and health changes during long life, the sampling procedure was directed to a permanently open recruitment, allowing continuous growth for an ever-increasing sample and no minimum sample size was determined. To this purpose, each year, all first-year and first-enrolment students of the 11 collaborating Spanish universities (Alicante, Cantabria, Castilla-La Mancha, Granada, Huelva, Jaén, León, Salamanca, Valencia, Valladolid, and Vigo) were invited to participate in the project through an institutional mailing.

The information was collected online through the platform SphinxOnline^®^ (v. 4.19, Le Sphinx Développement SARL, Annecy, France). This software kept data confidential, and it complied with Spanish Law 3/2018 on Data Protection. After accepting the informed consent form, they would answer the questionnaire, which takes approximately 30–45 min to complete.

### 2.2. Study Variables and Instruments

The self-administered uniHcos questionnaire includes 453 items on different areas: sociodemographic characteristics, habits and lifestyles, the diet, and substance use. This questionnaire was made from different validated surveys [35]. Additionally, since 2020, it includes a COVID-19 section with 18 more items.

The sociodemographic variables considered were sex, age, residence, people the student lived with, and field of knowledge. Self-reported body weight and height were considered to calculate the body mass index (BMI) of the participants. Four categories were made for this variable according to the BMI obtained: <18.5 kg/m^2^ underweight, 18.5–24.9 kg/m^2^ normal, 25–29.9 kg/m^2^ overweight, and >30 kg/m^2^ obesity. For some analyses, this variable was categorized as a dichotomous variable (<25 kg/m^2^, ≥25 kg/m^2^). These classifications were chosen according to the criteria established in recent studies on university populations [36].

Physical activity (PA) was assessed with the International Physical Activity Questionnaire-Short Form (IPAQ-SF). This questionnaire is composed of 7 questions and has a validated version in Spanish with a university population [37,38]. It identifies the total minutes over the last 7 days spent on moderate and vigorous-intensity PA, walking, and inactivity. The IPAQ-SF sum score is expressed in metabolic equivalent task (MET)-minutes per week. In the present study, in order to categorise participants according to their physical activity intensity, the results were dichotomised according to whether or not they meet the WHO criteria for physical activity [14]. 

Sedentary behaviour was also assessed by exploring the amount of time spent sitting per day, using the IPAQ-SF. This variable was divided into 4 categories: <4 h/day, 4–5 h/day, 6–8 h/day, and >8 h/day. For the dichotomous cut-off point, a sitting time of ≥7 h per day was established. These classifications were made along the lines of recent studies [39,40]. 

The diet variable was constructed from the answer to the food frequency consumption section (FFCS) of the online self-questionnaire, which was modelled after question 96 of Section H4 of the 2006 Spanish National Health Survey [35]. The FFCS has five options (daily; 3–4 times per week, but not daily; 1–2 times per week; <1 time per week; never/almost never) for the frequency of consumption of fruits; meat; processed meat (hamburgers, hot dogs, …); eggs; fish; pasta, rice, and potatoes; bread and grains; pizza; vegetables; pulses; sausages and cold meats; dairy; sweets; sugary drinks; and juices and milkshakes. Our data were adapted to the Healthy Eating Index (HEI–2015) [41] according to the recommendations of the Spanish Society of Community Nutrition [42]. For this purpose, a maximum score of 10 points and a minimum score of 0 according to the frequency of consumption was awarded to each category, so the maximum total score was 100. In this study, on the basis of the total score, two categories were defined: ≤80 the diet needs improvements/poor diet and >80 good diet/healthy diet. In addition, we also explored the participants who performed “skipping breakfast” and it was categorised as a dichotomous variable (Yes/No). 

From 2012 to 2019, questionnaires were categorized by the year in which the survey was filled out, and this period was defined as Before COVID-19 (BC). Questionnaires completed from May 2020 to May 2021 were included in a single period called Lockdown (LD). This period was established in accordance with the different Royal Decrees carried out for this purpose in Spain and its extensions [43,44], which established the imposition of an “Alarm State” throughout the Spanish territory, implying severe mobility and social restrictions, since the derogation of the “Alarm State” on 4 May [45]. Finally, the questionnaires completed from June 2021 to May 2022 were included in the period defined as New Normal (NN). 

### 2.3. Data Analysis

Data analyses were conducted using STATA version 17 [46]. A descriptive analysis was performed on the prevalence rate of the different categories of BMI over time and in relation to different variables, such as sex, physical activity, and the diet, and focusing on the possible impact of COVID-19. 

An analysis of descriptive data was performed using the mean and standard deviations (SD) for the quantitative variables, whereas frequency and percentages were used for the qualitative variables. A Pearson chi-square analysis was carried out to assess statistically significant differences between qualitative variables. To establish the association between BMI and different lifestyle habits, a logistic regression model was performed, and it was adjusted for age, sex, cohabitants, physical activity, sitting time, the diet, and skipping breakfast. For all analyses, the level of statistical significance was set at a *p*-value of ≤ 0.05.

### 2.4. Ethical Aspects

This work was carried out in agreement with the 2013 Helsinki Declaration. In addition, all ethics committees approved this study from the outset and have periodically renewed it, and the current code of ethics is ETICA-ULE-031-2020. Students participated freely without compensation of any kind and were made aware of the purpose of this study through informed consent.

## 3. Results

Table 1 shows the sociodemographic characteristics of the sample. The mean age of the students (standard deviation) was 19.0 years (1.5). The majority of participants were women (73.2%). Most students lived in the family household (46.0%) or in a rented apartment (39.7%), and their cohabitants were mainly their families (47.2%) or roommates/friends (44.7%). The participants came from five different branches of knowledge, the most predominant of which were social sciences (39.6%) and health sciences (22.3%).

### 3.1. Prevalence of BMI Categories According to Different Variables 

Table 2 shows the differences in BMI according to different variables. The majority of participants (72.9%) were within the normal weight range. 12.5% of females had low weight compared to 5.7% of males; on the other hand, 17.7% of men were overweight compared to 11.8% of women. Finally, more men also exhibited obesity than women (3.8% and 2.8%, respectively, *p* < 0.001). According to physical activity, the students who did not meet the WHO criteria had a higher underweight (12.4%), overweight (13.4%), and obesity (4.2%) prevalence compared to those who meet the criteria (10.2%, 13.3%, and 2.8%, respectively, *p* < 0.001). 

Considering sitting time, it is observed that the group with the highest percentage of students with normal weight was the one that spent less than 4 h per day sitting (74.1%). On the other hand, those who spent more than 8 h per day sitting were the ones who had the highest obesity rates (3.9%; *p* = 0.015).

Looking at the diet, it can also be seen that the prevalence of normal weight was higher in the group of people who followed a healthy diet, while many students with low weight followed an unhealthy diet or one that needs changes (*p* = 0.015). Finally, there were more students with normal weight who had breakfast (73.2%) and more individuals with obesity who skipped it (5.2 vs. 2.9%; *p* < 0.001).

### 3.2. Relation of BMI and Different Lifestyle Habits

Table 3 shows the results of the adjusted logistic regression model for the relation between BMI and different lifestyle habits. The regression model identified that age (*p* < 0.001), sex (*p* < 0.001), and living alone (*p* = 0.013) were statistically associated with an increased risk of obesity. In relation to lifestyle habits, the model revealed that people who did not meet WHO physical activity criteria had more risk of obesity *(p* = 0.029), as well as those who were sedentary *(p* = 0.015) or skipped breakfast (*p* = 0.004). 

### 3.3. Differences in BMI by Period of Study and Sex

**Figure 1:**
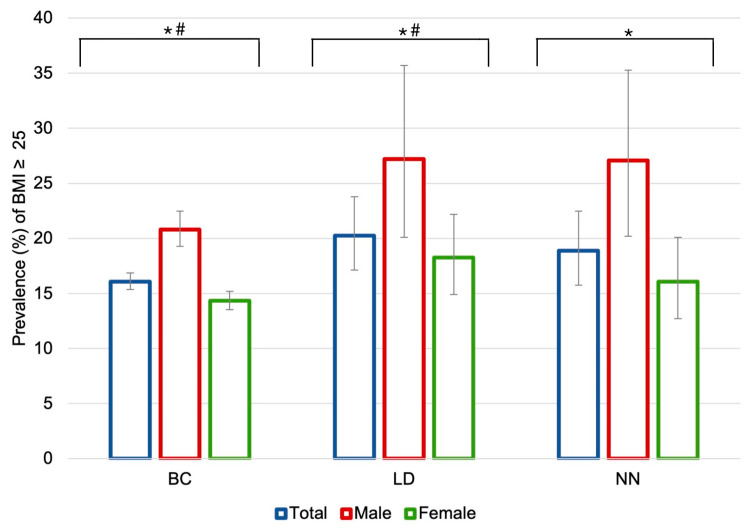
Differences in prevalence of BMI ≥ 25 kg/m^2^ in university students by period of study and disaggregated by sex. Error bars show the 95% CI. The symbol * indicates statistically significant differences (*p* < 0.05) between males and females in the same period. The symbol # indicates statistically significant differences (*p* < 0.05) between the whole samples of the different periods indicated (BC vs. LD). Abbreviations: BMI: body mass index; BC: Before COVID-19; LD: Lockdown; NN: New Normal.

Figure 1 shows the prevalence of overweight or obesity (BMI ≥ 25 kg/m^2^) in the three study periods (BC, LD, and NN). It can be seen that during the LD period, there was a statistically significant increase in the prevalence of BMI ≥ 25 kg/m^2^ (20.2, 95% CI: 17.1–23.8) compared to the previous period (16.1, 95% CI: 15.4–16.9). According to sex, in all periods, males had a higher prevalence rate of BMI ≥ 25 kg/m^2^ than females (*p* < 0.05). For both sexes, an upward trend is observed during the LD period, but in men, during the NN period, the prevalence of overweight remained stable (LD: 27.2%, 95% CI: 20.2–35.7; NN: 27.1%, 95% CI: 20.2–35.3), in women, there was a slight decrease (LD: 18.3%, 95% CI: 14.9–22.2; NN: 16.1%, 95% CI: 12.7–20.1).

### 3.4. Differences in Lifestyle Habits by Period of Study and Sex 

**Figure 2:**
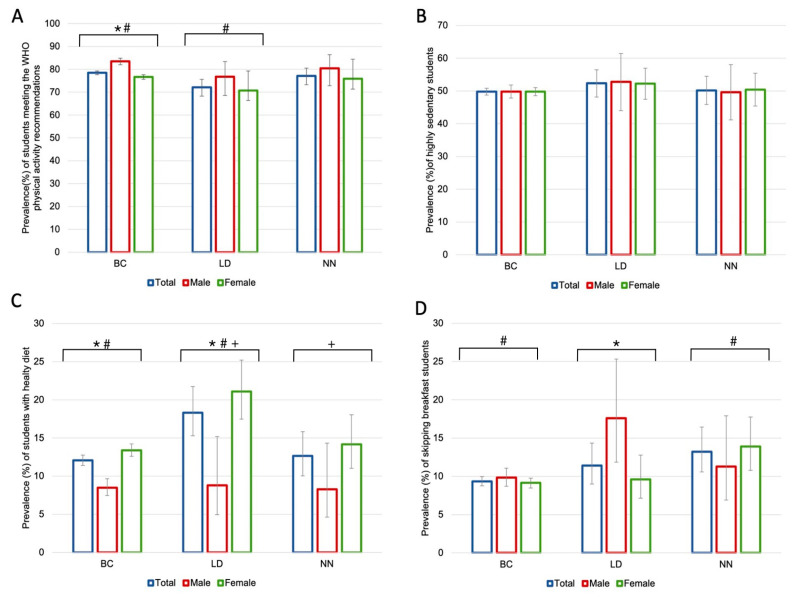
Changes in physical activity, sedentary behaviour, healthy diet, and skipping breakfast by period and sex. Error bars show the 95% CI. The symbol * indicates statistically significant differences (*p* < 0.05) between males and females in the same period. The symbols # and + indicate statistically significant differences (*p* < 0.05) between the whole samples of the different periods indicated (#: BC vs. LD in (**A**,**C**); #: BC vs. NN in (**D**); and +: LD vs. NN in (**C**)). Abbreviations: BC: Before COVID-19; LD: Lockdown; NN: New Normal; WHO: World Health Organization.

Figure 2 shows the prevalence of different lifestyle habits (physical activity, sedentary lifestyle, a healthy diet, and skipping breakfast) in the three study periods and disaggregating the data by sex. According to Figure 2A, the percentage of students who meet the WHO recommendations for physical activity was minor during the LD (BC: 78.7%, 95% CI: 77.7–79.3; LD: 72.4%, 95% CI: 68.3–75.7), and then increased again during the NN (77.1%, 95% CI: 73.3–80.5). During the BC period, the percentage of men who engaged in physical activity was higher than that of women (males: 83.5%, 95% CI: 82.0–84.9; females: 76.6%, 95% CI: 75.6–77.6), but these differences disappeared during Lockdown. Similar trends over time can be seen for both sexes.

Considering sedentary behaviour (Figure 2B), no statistically significant differences were observed between any of the periods, nor when studying differences between sexes. During the LD period, there was a higher percentage of students who spent 7 or more hours sitting, and during the NN, the percentage was lower again.

According to Figure 2C, the prevalence of students with a healthy diet was significantly greater during the LD (BC: 12.1%, 95% CI: 11.4–12.8; LD: 18.3%, 95% CI: 15.3–21.8) and minor during the NN (12.7%, 95% CI: 10.0–15.8). Both in the BC and LD periods, the prevalence of women with a healthy diet was higher (BC–males: 8.5%, 95% CI: 7.5–9.7; BC–females: 13.4%, 95% CI: 12.6–14.2; LD–males: 8.8%, 95% CI: 4.9–15.2; and LD–females: 21.1%, 95% CI: 17.5–25.2).

Finally, during the NN period (13.2%, 95% CI: 10.6–16.4), there was an increase in the percentage of students who fasted compared to the BC period (9.3%, 95% CI: 8.8–10.0), with no differences found between sexes. However, during the LD period, there was a higher prevalence of males than females who fasted (Figure 2D).

## 4. Discussion

This work had two main objectives; on the one hand, to study the possible relationship between BMI and different lifestyle habits, and on the other hand, to explore the possible impact of the COVID-19 pandemic on BMI and these health-related lifestyle habits. The obtained results indicate that overweight and obese students were more prone to skipping breakfast and to spending more time sitting, while the COVID-19 lockdown resulted in higher BMI values, lower physical activity levels, and better nutritional habits. The original approach of this investigation (it provides information on pre-, during-, and post-COVID-19 prevalence of healthy habits among freshmen), and the novelty of the findings, can help to expand the existing evidence in this regard. 

Most of the participants in this study showed a normal weight—a finding previously observed among freshmen studies [47]. Nevertheless, it is worth mentioning that around 20% of male and 15% of female students were overweight/obese. These values are lower than those observed among university students worldwide [9], and lend force to the “Freshmen 15” concept that implies that the rate of overweight/obesity increases through the university years [48]. In relation to this, it is worth mentioning that reductions in PA appear to be the defining characteristic in freshman weight gain [49].

An important aspect of the present study is that it confirms the relationship between being physically active and having a normal weight. Those students who meet the WHO criteria had a lower risk of being underweight, overweight, or obese. This was also the case for sedentary behaviour, as assessed through sitting time. These findings expand the idea previously reported that students with low PA levels are more likely to have excess body weight [36]. On a final note, it should also be mentioned that a healthy diet was associated with having a normal weight, while skipping breakfast was an unhealthy habit more frequently present among individuals with obesity. Associations among adolescents or emerging adults between skipping breakfast and overweight/obesity status have already been found in the literature [50], implying the need for developing strategies to promote healthy dietary habits in the university context.

In this research, we provide original information regarding the impact of three time periods (pre-, during, and post-COVID-19) on BMI values. Our findings indicate that during Lockdown, BMI ≥ 25 kg/m^2^ increased significantly, which is in agreement with previous studies indicating a rise in BMI values among university students during this period [51,52]. A novel finding is that in the NN, overweight status was reduced among females but not among male students. Studies have shown that female university students have higher body dissatisfaction levels than males [53]. Therefore, it could be speculated that women who answered the questionnaire during the NN were more worried about the changes in their body image as a result of the Lockdown period and tried to lose weight, while men did not follow this tendency. This idea is in agreement with the findings of Duan et al. [54], who reported that almost 20% of the Chinese university students who were surveyed during the post-lockdown era were overweight/obese with a higher rate in males. Other authors have observed mixed responses, with university students indicating that either they had put on or they had lost weight during the post-lockdown period [55]. Some students even reported that they were not concerned about their weight, as previously speculated. These results are somewhat challenging to compare because information on educational paths and assistance in reintegrating into a regular lifestyle is often not precisely reported in these types of studies.

According to our findings, the prevalence of PA levels during the Lockdown period was significantly less than Before COVID-19, which could indicate a reduction in the practice of PA as found in investigations focused on university students [56]. A tendency worth mentioning regarding PA levels is that while males were more active than females in the pre-COVID-19 period, which is an expected finding [57], these differences disappeared during Lockdown. This result agrees with the idea that those university students who were more active were the ones whose PA levels were more impacted by the imposed quarantine period [58]. We could not find any investigation in which the PA levels of university students before, during, and after the COVID-19 quarantine were analysed. This fact hampers further discussion; however, it provides new information on how students regain healthy habits after a period of imposed transition. This information makes it possible to develop preventive educational interventions to anticipate future crises.

There are some studies that have provided information on other population groups and that reported similar findings. For instance, Mc Carthyl et al. [59] tracked PA before, during, and after the United Kingdom’s COVID-19 lockdown, and found a general decrease in PA prevalence, influenced by age. Specifically, young people were the group that showed lower PA levels while those older than 65 years appeared to remain more active as soon as the lockdown was eased. In a longitudinal study carried out in New Zealand, Hargreaves et al. [60] reported that compared to pre-lockdown, total PA was lower during and post-lockdown for highly active individuals, while those who were moderately active pre-lockdown increased their PA levels during the lockdown and maintained them post-lockdown. All together, these findings confirm that the lockdown had a greater impact on those individuals who were more active. This is not surprising since these individuals are the ones who showed higher levels of physical activity prior to the pandemic. In any event, data from the NN phase indicate an increase in PA prevalence of female and male students until both reached satisfactory PA levels, which seems to be in agreement with the PA prevalence found among university students worldwide [61].

Several authors have confirmed that among university students, sedentary behaviour increased due to the lockdown period [62,63]. In the present investigation, this trend was also observed, albeit no statistical differences were observed when the three time periods were analysed. A similar tendency was observed by ten Velde et al. [64], who gathered information on sedentary behaviour as assessed through screen time pre-, during, and post-school closures of Dutch children. Their results indicated that screen time increased during the lockdown and was still fairly present during the NN. In the end, our findings point out that sedentary time was higher during Lockdown in comparison with the previous period, and lend force to the idea that during the NN, levels of sedentary behaviour were reduced. This is an important fact to consider, since sedentary levels are fairly present among university students. Moreover, it has been stated that this population may be both highly active and highly sedentary [65], an observation that can help to explain the findings shown here.

In relation to eating habits, our findings indicate that students were more prone to follow a healthy diet during Lockdown, which confirms previous reports indicating that young adults improved the quality of their diet during this period, allegedly due to eating out less often [66]. This idea can help to explain why once the NN was established, healthy diet adherence decreased again. In this line, Zhang et al. [67] observed the prevalence of an unbalanced diet (notably a decrease in seafood consumption) in China in the post-lockdown period. An important result that should not be overlooked is that an increase in the percentage of university students who fasted was observed during this period, which agrees with previous investigations indicating a decrease in caloric intake among freshmen [32].

In this study, we add to the growing body of research on the health impact of the COVID-19 pandemic by comparing changes in healthy habits before, during, and after the lockdown period. In spite of the novelty of these findings, there are several limitations that should be acknowledged. In the first place, data were self-reported, thus caution is advised when interpreting the obtained results, especially data on BMI. Secondly, we did not follow the same cohort during the three study periods. Instead, we assumed that the samples were comparable to each other, as they had common characteristics (i.e., Spanish first-year university students), but it should be considered that due to a question of temporality, the sample sizes are different in the study periods. Finally, we should not rule out the existence of a possible selection bias, implying that only those students who were motivated participated in the survey. Thus, our sample might not be fully representative of the target population. However, it is hoped that this research will provide a basis for the development of future longitudinal studies within this project, as well as contribute to novel findings on lifestyle habits of university students.

## 5. Conclusions

This study shows that most of the freshmen had a normal weight and that lifestyles are associated with BMI, presenting an association between normal weight and healthy habits such as complying with the WHO recommendations on physical activity, while overweight and obesity were associated with skipping breakfast, spending more time sitting, being men, and living alone.

On the other hand, this study suggests that the COVID-19 pandemic and the subsequent LD measures had a significant impact on BMI and health-related lifestyle habits. An increase in BMI, a reduction in physical activity, and an increase in the prevalence of a healthy diet were observed during the Lockdown period—each lifestyle prevalence returned to levels close to those of the pre-COVID-19 period in the New Normal, which indicates the importance of the limitation of access in the habits of the student population. Differences by sex were observed in BMI prevalence and in changes in lifestyles among periods.

Based on these results, it seems clear that it is necessary to propose public health interventions that improve the lifestyles of university students and that consider the differences between the sexes and the relevant role of the availability of access to lifestyle, which has been shown to be an essential factor of the changes detected because of the COVID-19 pandemic and the restrictions carried out.

## Figures and Tables

**Table 1:** Sociodemographic characteristics of the participants.

	N	%
Total	10,096	100
Sex		
Female	7389	73.2
Male	2707	26.8
Period of study		
Before COVID-19	9030	89.4
Lockdown	552	5.5
New Normal	514	5.1
Residence		
Family household	4640	46.0
Rented apartment	4006	39.7
University hall of residence	1254	12.4
Other	196	1.9
People the student lives with		
Family	4765	47.2
Roommates/Friends	4509	44.7
Alone	822	8.1
Branch of Knowledge		
Social Sciences	3993	39.6
Health Sciences	2252	22.3
Science	1589	15.8
Art and Humanities	1229	12.2
Engineering and Architecture	1024	10.2

**Table 2:** Differences in physical activity, sitting time, diet, and skipping breakfast according to BMI.

BMI					*p*-Value
	<18.5 kg/m^2^	18.5–24.9 kg/m^2^	25–29.9 kg/m^2^	≥30 kg/m^2^	
	N (%)	N (%)	N (%)	N (%)	
Total	1080 (10.7)	7358 (72.9)	1347 (13.3)	311 (3.1)	
Sex					**<0.001**
Female	925 (12.5)	5386 (72.9)	869 (11.8)	209 (2.8)	
Male	155 (5.7)	1972 (72.8)	478 (17.7)	102 (3.8)	
Physical activity					**<0.001**
Meets WHO criteria	808 (10.2)	5824 (73.7)	1053 (13.3)	220 (2.8)	
Does not meet WHO criteria	272 (12.4)	1534 (70.0)	294 (13.4)	91 (4.2)	
Sitting time (hours/day)					**0.015**
<4	186 (9.9)	1393 (74.1)	243 (12.9)	58 (3.1)	
4–5	221 (10.7)	1526 (74.0)	270 (13.1)	45 (2.2)	
6–8	266 (10.7)	1821 (73.5)	327 (13.2)	64 (2.6)	
>8	407 (11.1)	2618 (71.2)	507 (13.8)	144 (3.9)	
Diet (adapted HEI)					**0.015**
>80 good	110 (8.8)	929 (74.0)	172 (13.7)	44 (3.5)	
≤80 needs improvement/poor	970 (12.7)	6429 (72.9)	1175 (10.9)	267 (3.5)	
Skipping breakfast					**<0.001**
Yes	98 (10.1)	684 (70.1)	142 (14.6)	51 (5.2)	
No	982 (10.8)	6674 (73.2)	1205 (13.2)	260 (2.9)	

*p*-value results in bold indicate statistically significant differences for the chi-square test. Abbreviations: BMI: body mass index; HEI: Healthy Eating Index; WHO: World Health Organization.

**Table 3:** Logistic regression model for relation between BMI ≥ 25 kg/m^2^ and different lifestyle habits.

BMI ≥ 25 kg/m^2^				
	OR (95% CI)	*p*-Value	aOR (95% CI)	*p*-Value
Age	**1.14 (1.11–1.18)**	**<0.001**	**1.14 (1.11–1.18)**	**<0.001**
Sex				
Female/Male	**0.60 (0.56–0.70)**	**<0.001**	**0.62 (0.55–0.69)**	**<0.001**
People the student lives with				
Roommates/Friends vs. Family	1.10 (0.98–1.22)	0.143	1.10 (0.98–1.23)	0.098
Alone vs. Family	**1.27 (1.69–1.99)**	**0.014**	**1.27 (1.05–1.55)**	**0.013**
Physical activity				
Does not meet WHO criteria vs. meets WHO criteria	1.11 (0.97–1.26)	0.101	**1.15 (1.01–1.31)**	**0.029**
Sitting time				
Highly sedentary vs. not	**1.05 (1.00–1.10)**	**0.039**	**1.06 (1.01–1.11)**	**0.015**
Diet (adapted HEI)				
Poor diet vs. healthy diet	0.93 (0.80–1.10)	0.420	0.87 (0.74–1.03)	0.092
Skipping breakfast				
Yes vs. No	**1.29 (1.09–1.52)**	**0.003**	**1.28 (1.08–1.52)**	**0.004**

*p*-values in bold indicate statistically significant results. aOR by age, sex, cohabitants, physical activity, sitting time, diet, and skipping breakfast. Abbreviations: aOR: adjusted odds ratio; CI: confidence interval; BMI: body mass index; HEI: Healthy Eating Index; WHO: World Health Organization.

## Data Availability

The datasets analysed in the current study are available from the corresponding author upon reasonable request.
